# Valproic Acid Reduces Invasiveness and Cellular Growth in 2D and 3D Glioblastoma Cell Lines

**DOI:** 10.3390/ijms26146600

**Published:** 2025-07-09

**Authors:** Francesca Giordano, Martina Forestiero, Adele Elisabetta Leonetti, Giuseppina Daniela Naimo, Alessandro Marrone, Francesca De Amicis, Stefania Marsico, Loredana Mauro, Maria Luisa Panno

**Affiliations:** 1 Health and Nutritional Sciences, Department of Pharmacy, University of Calabria, 87036 Rende, Italy; martina.forestiero@unical.it (M.F.); adeleelisabetta.leonetti@unical.it (A.E.L.); giuseppinadaniela.naimo@unical.it (G.D.N.); francesca.deamicis@unical.it (F.D.A.); stefania.marsico@unical.it (S.M.); loredana.mauro@unical.it (L.M.); 2 Ecology and Earth Sciences, Department of Biology, University of Calabria, 87036 Rende, Italy; alessandro.marrone@unical.it

**Keywords:** glioblastoma, valproic acid, EMT, apoptosis, ROS

## Abstract

Glioblastoma (GBM) is the most common malignant brain tumor, with a poor prognosis and low survival. Its treatment includes complete surgical resection followed by radiotherapy combined with temozolomide (TMZ). GBM contains glial stem cells (GSCs), which contribute to tumor progression, invasiveness, and drug resistance. The histone deacetylase (HDAC) inhibitor valproic acid (VA) has been shown to be a potent antitumor and cytostatic agent. In this study, we tested the effects of VA on glioma cell proliferation, migration, and apoptosis using T98G monolayer and spheroid cells. T98G and U-87MG glioblastoma cell viability was determined by MTT. Cell cycle and ROS levels were analyzed by flow cytometry, and gene and protein levels were detected, respectively, by RT-PCR and immunoblotting. VA reduces cell viability in 2D and 3D T98G and U-87MG cells and blocks the cell cycle at the G0/G1 with decreased levels of cyclin D1. VA addresses apoptosis and ROS production. In addition, VA significantly decreases the mRNA levels of the mesenchymal markers, and it counteracts cell migration, also decreasing MMP2. The results confirm the inhibitory effect of VA on the growth of the T98G and U-87MG cell lines and its ability to counteract migration in both 2D and 3D cellular models.

## 1. Introduction

Glioblastoma is the most common and aggressive malignant primary brain tumor thought to originate from glial cells. It is associated with a high morbidity and mortality rate [1]. In the most recent classification by the World Health Organization (WHO), published in 2021, gliomas were divided into four grades according to their histological and molecular characteristics, namely, Grade I to IV, with Grade IV, often called glioblastoma multiforme (GBM), being the most aggressive and malignant [2]. The updated classification of glioblastoma (2021 WHO) incorporates both histological and molecular features. Glioblastomas are classified as IDH-wildtype or IDH-mutant. The wildtype form is characterized by EGFR amplification, TP53 mutations, and PTEN loss, with frequent chromosomal abnormalities. MGMT promoter methylation is a key prognostic marker. IDH-mutant glioblastomas typically have IDH1/IDH2 mutations, TP53 mutations, ATRX loss, and are associated with a better prognosis [3].

GBM has a median survival of 15 months, and only 5.5% of patients survive for 5 years after being diagnosed [3]. According to histopathological assessments, glioblastoma is regarded as an aggressive and infiltrative tumor, characterized by high mitotic activity, necrosis, and significant vascularization, despite the lack of metastasis in other organs [4,5].

These characteristics contribute to the low survival rate, depending also on whether surgical tumor resection is successful, as well as on the tumor’s resistance to chemotherapy and radiotherapy [6,7].

Another important feature is the presence of the tumor’s own stem cells (GSCs), which are characterized by a high proliferative capacity and the ability to differentiate into different cell types resulting from the tumor’s cellular heterogeneity [8].

The presence of these cells is responsible for the recurrence of the GBM after surgical resection and therapeutic treatment. It also renders the cancer resistant to pharmacological therapies, which are already limited by the presence of the blood–brain barrier. Temozolomide is the first-line chemotherapy drug for the treatment of GBM. In addition, the combination of temozolomide with tumor-directed fields has been shown to increase median overall survival to 20.5 months compared with standard chemotherapy (15.6 months) [9].

In addition to conventional adjuvant chemotherapy, molecular therapies, immunotherapies, and their combined treatments are increasingly being explored as options for the management of GBM [10].

Growing interest in epigenetic mechanisms as therapeutic targets is supported by increasing evidence that epigenetic alterations, in conjunction with genetic changes, may significantly contribute to the development and/or progression of gliomas [11]. Epigenetic modifications, such as DNA methylation, histone methylation, histone acetylation, and non-coding RNA, play crucial roles in the regulation of gene expression and cellular function. Among these, histone acetylation has attracted significant attention due to its reversible nature and its impact on chromatin structure, making it a key focus for therapeutic intervention. Therefore, the reversibility of epigenetic alterations encourages the use of histone deacetylase inhibitors (HDACi) as an attractive approach to “reset” the abnormal cancer epigenome [12].

Valproic acid (VA, 2-propylpentanoic acid) is a short-chain fatty acid that has been in use worldwide for decades in the treatment of epilepsy and bipolar disorder [13]. It is one of the most commonly used histone deacetylase inhibitors (HDACI), which modulate histone deacetylases—enzymes involved in chromatin charge modification. This mechanism regulates several genes involved in cell cycle control and apoptosis [14,15].

VA is known to have anticancer activity in a variety of tumor cell lines, such as gastric, prostatic, and breast, either alone or in combination with other compounds [16,17,18,19].

VA also modulated the invasive ability of breast cancer cells by targeting MMP-1, MMP-3, and MMP-13, thereby enhancing the effectiveness of radiotherapy [20].

In glioblastoma cells, the combined treatment of VA with temozolomide (TMZ) has synergistic effects compared to chemotherapy alone since it serves as an adjuvant factor to sensitize the cells [21,22].

Meanwhile, VA contrasts the invasion potential of glioma cells by influencing cell polarity and the expression of the cell–cell adhesion proteins [23].

The goal of this study was to evaluate the biological effects of VA on T98G and U87MG glioblastoma cell lines in both monolayer and spheroid cellular models by addressing its role on cell growth, migration, and EMT process.

We used a 3D spheroid cell culture, a model that enables more physiologically cellular behavior, including cell–cell interactions, extracellular matrix deposition, and response to drugs, compared to traditional 2D cell cultures.

## 2. Results

### 2.1. Valproic Acid Reduces Cell Viability and Migration of Glioblastoma Cells

First, we investigated the effects of increasing doses (1, 2, 4, 6, 8, and 10 mM) of the valproic acid (VA) for 24, 48, and 72 h on the viability of T98G and U-87MG glioblastoma cell lines using an MTT assay.

**Figure 1:**
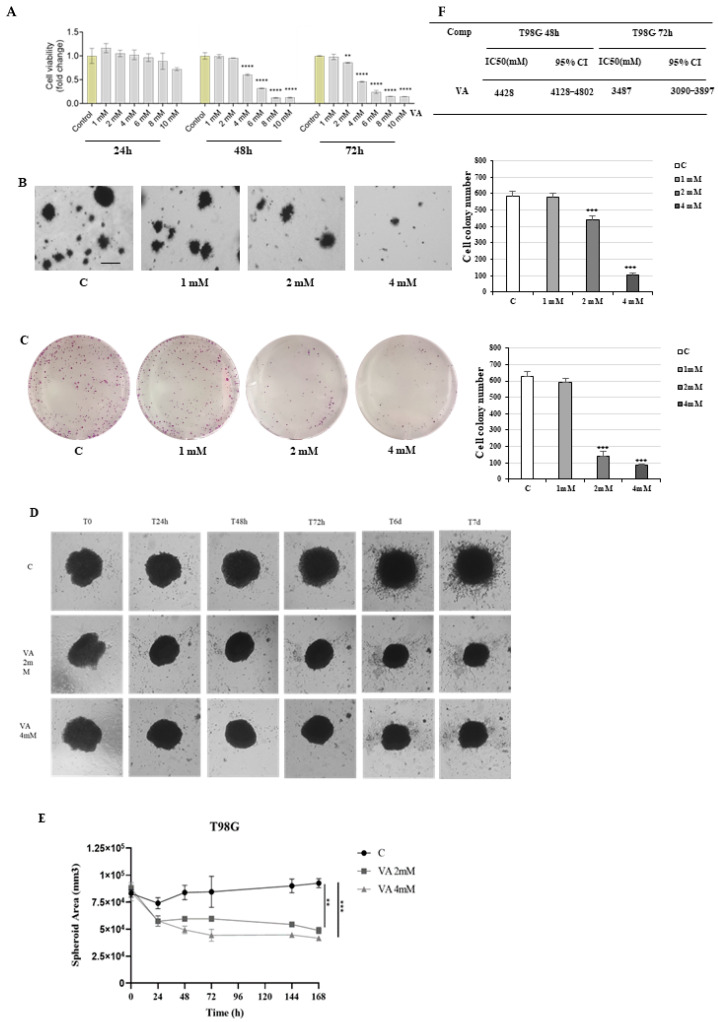
Effects of VA on the growth of glioblastoma cells. (**A**) Effect of valproic acid on cell survival in human glioblastoma cells, T98G, incubated for 24, 48, and 72 h. The data are the mean of *n* = 3 determinations performed in triplicate. The data are presented as mean ± SEM, evaluated by one-way ANOVA and Dunnett’s multiple comparison tests: ** *p* < 0.01, **** *p* < 0.0001. IC50 (half-maximal inhibitory concentration) of VA for T98G glioblastoma cancer cells. CI, confidence interval (**F**). (**B**) Representative images of soft agar-grown colonies of T98G glioblastoma cells, either untreated (control, C) or treated with VA 1 mM, 2 mM, and 4 mM for 14 days (4× magnification). Scale bar: 50 µM. The histogram shows the numbers of colonies formed per well. Values are representative of three independent experiments, each performed in triplicate. *** *p*  <  0.001 vs. C (Student’s *t*-test). (**C**) Representative images of colony formation assay in T98G glioblastoma cells, wither untreated (control, C) or treated with VA 1 mM, 2 mM, and 4 mM. After 14 days of incubation, cell colonies were stained, and images were captured by a digital camera. Quantification of cell colony number using Image J software. Each data point is the mean ± SD of three independent experiments performed in triplicate. *** *p*  <  0.001 vs. C (Student’s *t*-test). (**D**) Comparison of T98G spheroids’ growth inside collagen I and Matrigel formed on a 24-well plate, treated in white medium, control, or with 2 mM and 4 mM of VA. (**E**) Graph of spheroid area monitored over time. Each group is compared to the control group. Data represents mean ± SD from independent measurements, four wells per condition. ** *p* < 0.01 and *** *p* < 0.001 (Student’s *t*-test).

**Figure 2:**
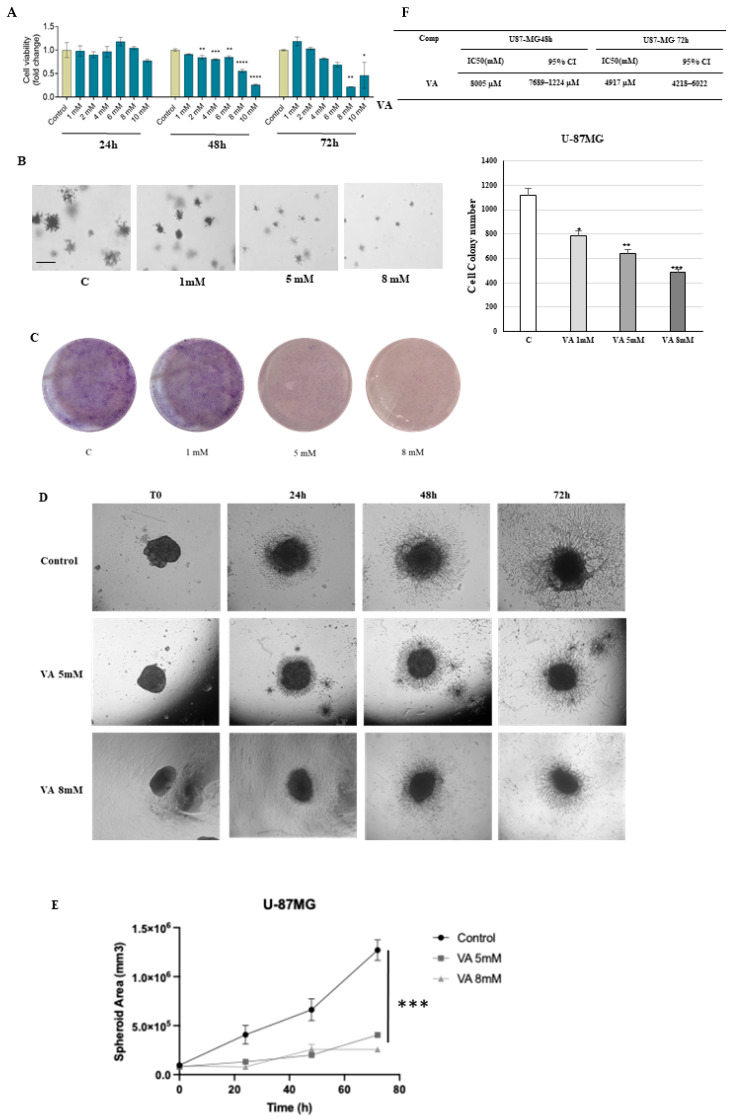
Effects of VA on thew growth of glioblastoma U-87MG cells. (**A**) Effect of valproic acid on cell survival in human glioblastoma cells, U-87MG, incubated for 24, 48, and 72 h. The data are the mean of n = 3 determinations performed in triplicate. Data presented as mean ± SEM, evaluated by one-way ANOVA and Dunnett’s multiple comparison tests: * *p* < 0.05, ** *p* < 0.01, *** *p* < 0.001, **** *p* < 0.0001. IC50 (half-maximal inhibitory concentration) of VA for U-87MG glioblastoma cancer cells. CI, confidence interval (**F**). (**B**) Representative images of soft agar-grown colonies of U-87MG glioblastoma cells, either untreated (control, C) or treated with VA 1 mM, 5 mM, and 8 mM for 10 days (4× magnification). Scale bar: 50µm. The histogram shows the numbers of colonies formed per well. Values are representative of three independent experiments, each performed in triplicate * *p* < 0.05, ** *p* < 0.01, *** *p*  <  0.001 vs. C. (**C**) Representative images of colony formation assay in T98G glioblastoma cells, either untreated (control, C) or treated with VA 1 mM, 5 mM, and 8 mM. After 10 days of incubation, cell colonies were stained, and images were captured by a digital camera. (**D**) Comparison of U-87MG spheroids’ growth inside collagen I and Matrigel formed on a 24-wel plate, treated in white medium, control, or with 5 mM and 8 mM of VA. (**E**) Graph of spheroid area monitored over time. Each group is compared to the control group. Data represents mean ± SD from independent measurements, four wells per condition. *** *p* < 0.001 (Student *t*-test).

The results showed a dose- and time-dependent reduction in the viability of T98G cells compared to control (Figure 1A). The half-maximal inhibitory concentration (IC 50) values for the VA are shown in Figure 1F. These findings were further supported by similar observations in the human glioblastoma cell line U-87MG (Figure 2A,F). In SVGp12 cells, a human fetal glial cell line derived from normal human astroglial cells, VA treatment at doses ranging from 1 to 8 mM for 24 and 48 h did not reduce cell viability (Figure S1). These findings support the hypothesis that VA impairs cell viability selectively in tumor cells while sparing non-tumor glial cells

Next, we examined the effects of VA on the clonogenic potential of glioblastoma cells using two different assays: the soft agar growth assay to assess anchorage-independent growth, and the standard colony formation assay on adherent plates. In both assays, T98G and U-87MG cells were treated with VA and allowed to grow for 14 days and 10 days, respectively. Consistent with the MTT results, VA treatment significantly reduced both the number and size of colonies formed by T98G and U-87MG cell types (Figure 1B,C and Figure 2B,C).

To overcome the limitations of the 2D cell culture, which does not mimic the natural architecture of the cellular tissues, a 3D culture model of GBM was applied in the current study to examine the drug’s effects, and we compared the results with those of monolayer cells.

To this end, we performed a growth study of T98G and U-87MG cells seeded on Matrigel, a gold-standard gelatinous protein derived from the basement membrane, by using the same VA concentrations as those tested in the monolayer cultures. The results shown in Figure 1D,E and Figure 2D,E display the reduced growth and size of the 3D T98G- and 3D U-87MG-treated cells in a dose- and time-dependent manner.

**Figure 3:**
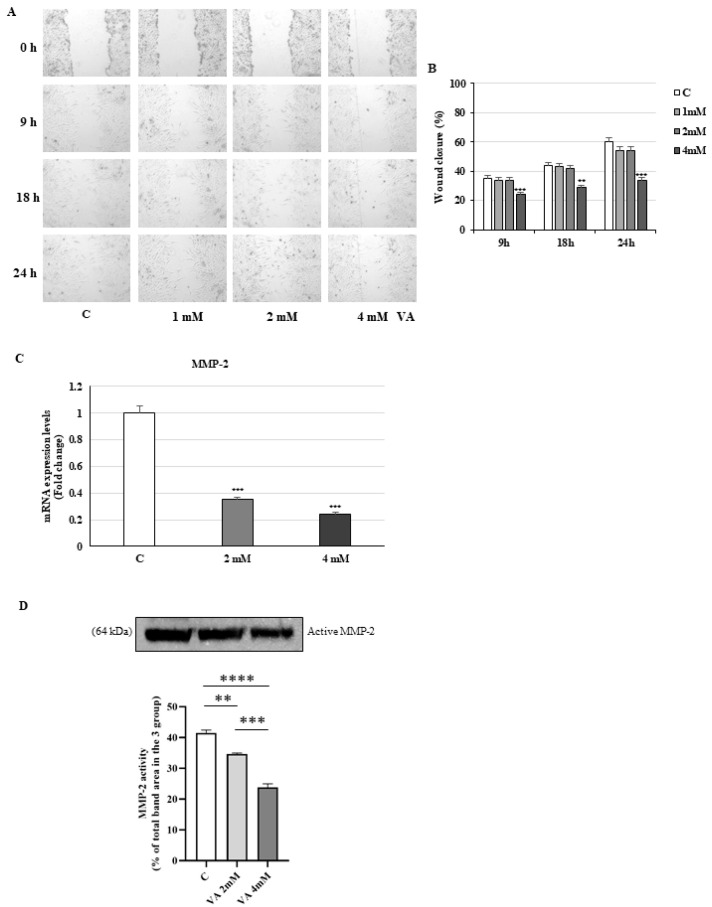
Effects of VA on cell migration. (**A**) Representative images from in vitro scratch wound healing assays in T98G glioblastoma cells, either untreated (control, C) or treated with VA 1 mM, 2 mM, and 4 mM (4× magnification). (**B**) The histograms, illustrating wound closure rates at indicated time points during the scratch wound assay (9 h, 18 h, and 24 h), represent the means ± S.D. of three different experiments, each performed in triplicate. Data were analyzed by Student’s *t*-test using the GraphPad Prism 7 software program. ** *p* < 0.01, *** *p* < 0.001 vs. C. (**C**) The mRNA levels of MMP-2 were evaluated by qRT-PCR using specific primers. The 18S mRNA levels were determined to normalize each sample. The histogram represents the mean ± S.D. of three distinct experiments, and the values are represented as fold changes compared to the control set, equal to 1. *** *p* < 0.001 vs. C. (**D**) Gelatin SDS-PAGE zymographic analysis of intracellular MMP-2 activity in T98G glioblastoma cells. Data are expressed as the mean ± SEM (n = 3 independent experiments). Significant differences were detected by one-way ANOVA and Newman–Keuls multiple comparison tests. ** *p* < 0.01; *** *p* < 0.001; **** *p* < 0.0001.

Since glioblastoma cells are known to be a highly invasive and infiltrative, the potential antimigratory effect of the drug was also evaluated in 2D T98G cells using the wound healing assay. In untreated cells, after 9 h, cells migrated to close the gap and reduced the surface area of the scratch. In contrast, cells treated with VA showed a significant decrease in migratory capacity over the treatment time (Figure 3A,B).

In agreement with these data, i.e., the mRNA levels and the zymography analysis of intracellular MMP2, the protein with the property of IV collagen degradation was reduced by VA (2 mM and 4 mM) after 24 h of treatment (Figure 3C,D).

**Figure 4:**
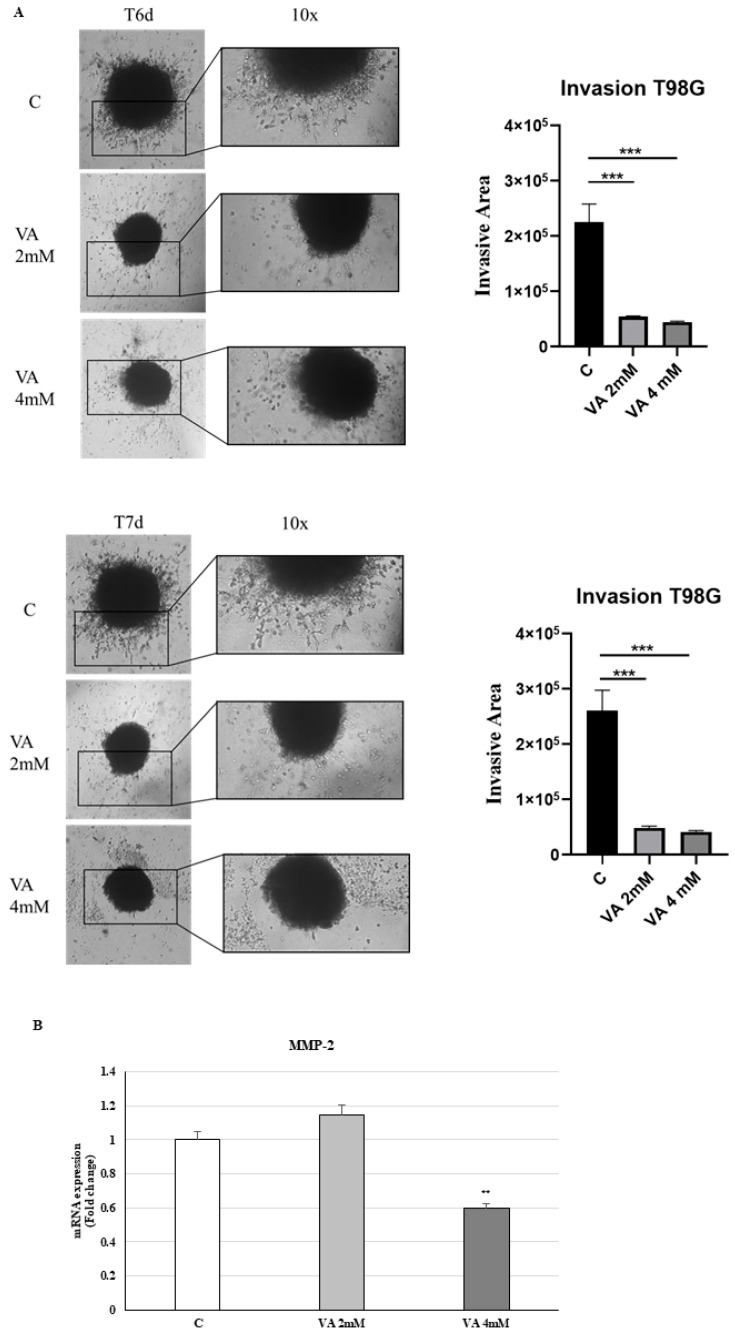
Time-lapse images of spheroid invasion assay inside collagen I and Matrigel. (**A**) Comparison of T98G spheroids formed on a 24-well plate, treated in white medium, control, or with 2 mM and 4 mM of VA (left panel). Graph of invasive area monitored on days 6 and 7. Each group is compared to the control group (right panel). Data represents mean ± SD from independent measurements, with four wells per condition. *** *p* < 0.001. (**B**) The mRNA levels of MMP-2 were evaluated in VA-treated glioblastoma cells for 24 h by qRT-PCR using specific primers. The 18S mRNA levels were determined to normalize each sample. The histograms represent the mean ± S.D. of three distinct experiments, and the values are represented as fold changes compared to the control set, equal to 1. ** *p* < 0.01.

We then performed in vitro invasiveness assay (inside collagen I and Matrigel) of 3D glioblastoma cells. Microscopy magnification images at day 6 and day 7 documented the spreading behavior of 3D T98G control cells, a process that was clearly arrested by VA at both concentrations used (Figure 4A). We observed a significative decrease in the mRNA level of MMP-2 at the highest concentrations of VA (Figure 4B).

**Figure 5:**
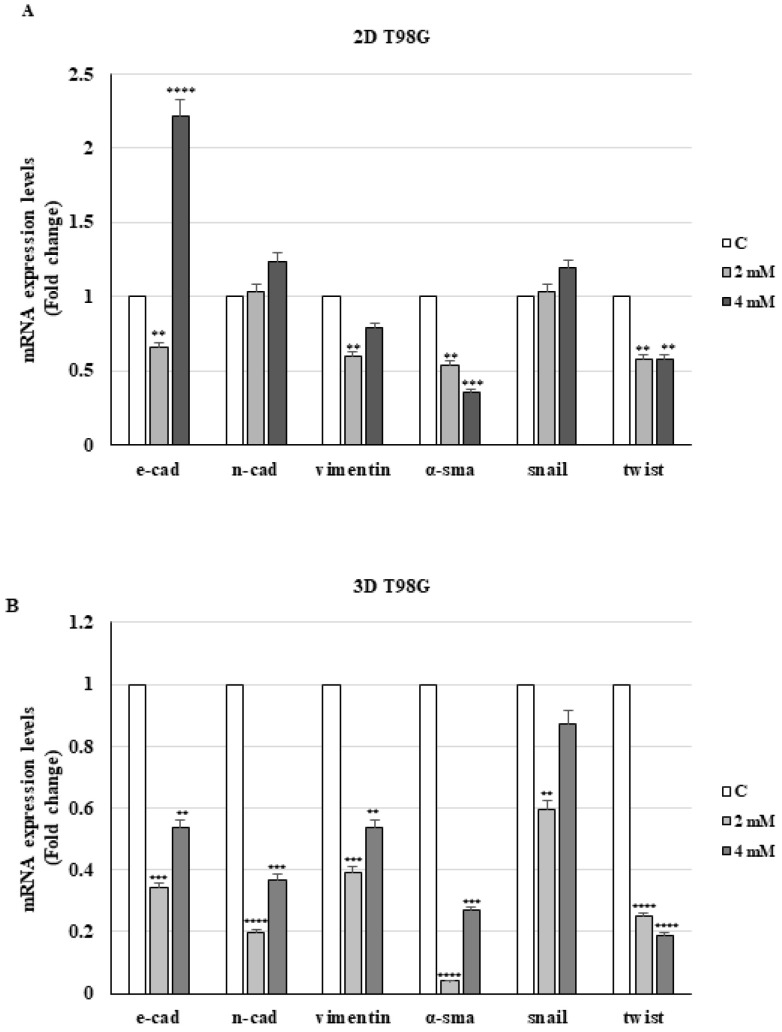
mRNA expression of E-cadherin, N-cadherin, vimentin, α-SMA, snail, and twist in 2D (**A**) and 3D (**B**) T98G cells. The mRNA levels were evaluated after 24 h of treatment with VA by qRT-PCR using specific primers. The 18S mRNA levels were determined to normalize each sample. The histograms represent the mean ± S.D. of three distinct experiments, and the values are represented as fold changes compared to the control set, equal to 1. ** *p* < 0.01 *** *p* < 0.001 vs. C **** *p* < 0.0001.

In a like manner, in both 2D and 3D culture cells, we evaluated whether the drug treatment might affect the epithelial–mesenchymal transition (EMT), which plays an important role in tumor progression and invasiveness. To this end, we examined the gene expression linked to this mechanism. The results indicated that VA in 2D T98G cells significantly decreased the expression of the mRNA levels of mesenchymal markers such as vimentin, α-SMA, and twist, while VA at the dose of 4 mM increased the E-cadherin mRNA, a well-known epithelial marker, and the N-cadherin (Figure 5A).

**Figure 6:**
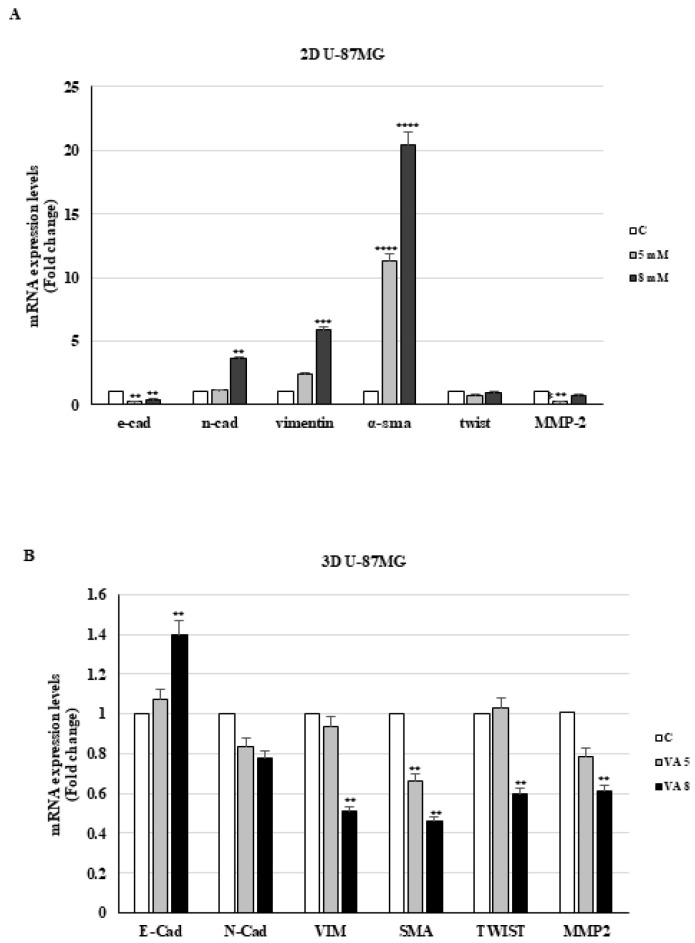
mRNA expression of E-cadherin, N-cadherin, vimentin, α-SMA, twist, and MMP-2 in 2D (**A**) and 3D (**B**) U-87MG cells. The mRNA levels were evaluated after 24 h of treatment with VA by qRT-PCR using specific primers. The 18S mRNA levels were determined to normalize each sample. The histograms represent the mean ± S.D. of three distinct experiments, and the values are represented as fold changes compared to the control set, equal to 1. ** *p* < 0.01 *** *p* < 0.001 vs. C **** *p* < 0.0001.

In contrast, valproic acid significantly upregulated the mRNA levels of mesenchymal markers and downregulated E-cadherin and MMP-2 mRNA expression in 2D U-87MG cells relative to the control (Figure 6A).

In VA-treated 3D T98G cells, we observed a more significant lowering in mRNA levels of specific mesenchymal genes, compared to cells grown in 2D (Figure 5B). In accordance with what was just outlined, the levels of E-cadherin and N-cadherin are also decreased by the drug. Unlike in U-87MG cell monolayers, valproic acid decreases mesenchymal marker expression and upregulates E-cadherin mRNA in spheroids derived from the same cells (Figure 6B).

These data on the 3D culture model of GBM strongly support the antagonistic effects of VA on tumor mass growth and provide additional evidence for the inhibition of cell invasiveness.

### 2.2. Effects of VA on Glioblastoma Cell Cycle

**Figure 7:**
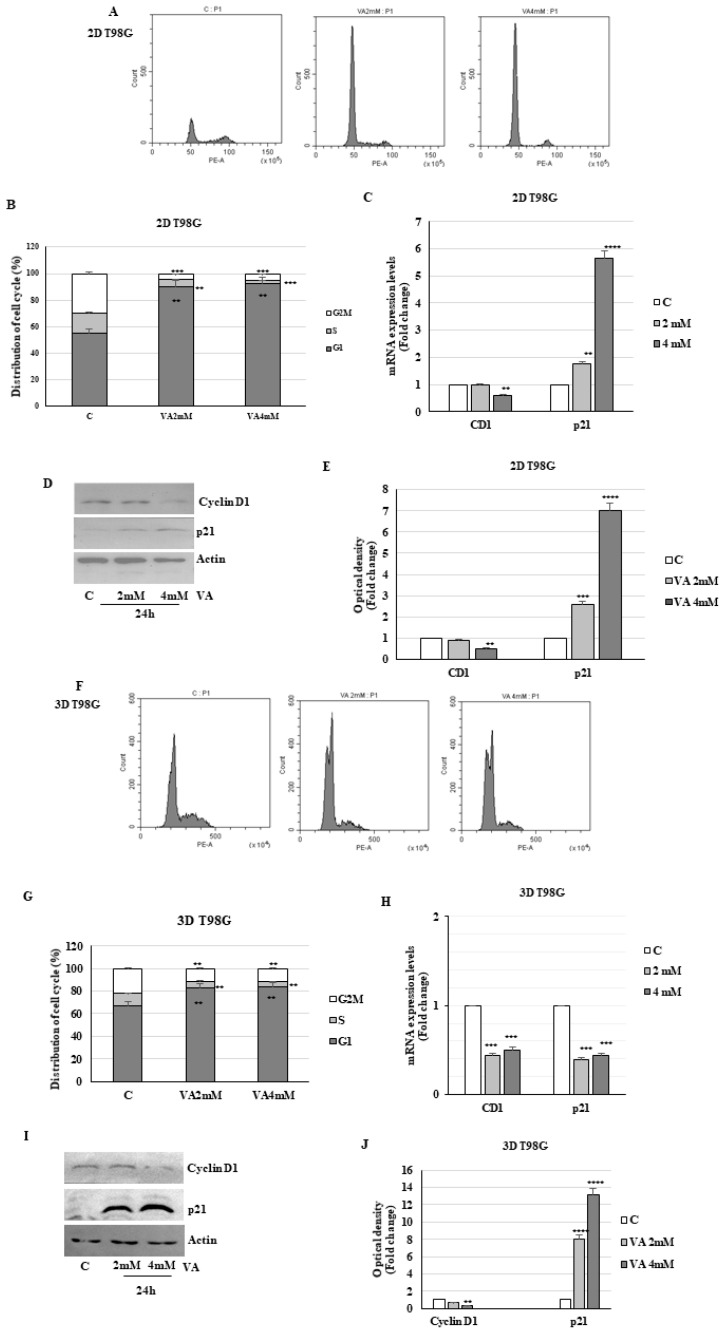
Action of VA on cell cycle in T98G glioma cells. Two-dimensional (**A**) and three-dimensional (**F**) T98G cells were treated with VA, as indicated, for 24 h, stained with propidium iodide (PI), and analyzed on a flow cytometer. Quantitative analyses of percentage gated cells at G0/G1, S, and G2/M phases are shown (**B**,**G**). The histograms represent the mean ± S.D. of three distinct experiments. ** *p* < 0.01, *** *p* < 0.001. The mRNA levels of cyclin D1 and p21 were evaluated by qRT-PCR using specific primers (**C**,**H**). The 18S mRNA levels were determined to normalize each sample. The histograms represent the mean ± S.D. of three distinct experiments, and the values are represented as fold changes compared to the control set, equal to 1. ** *p* < 0.01, *** *p*< 0.001, **** *p* < 0.0001 vs. C. Protein levels of cyclin D1 and p21 were analyzed by Western blot (**D**,**I**). The histograms represent the mean ± S.D. of three distinct experiments, and the values are represented as fold changes compared to the control set, equal to 1 (**E**,**J**). ** *p* < 0.01 *** *p* < 0.001 vs. C **** *p* < 0.0001.

**Figure 8:**
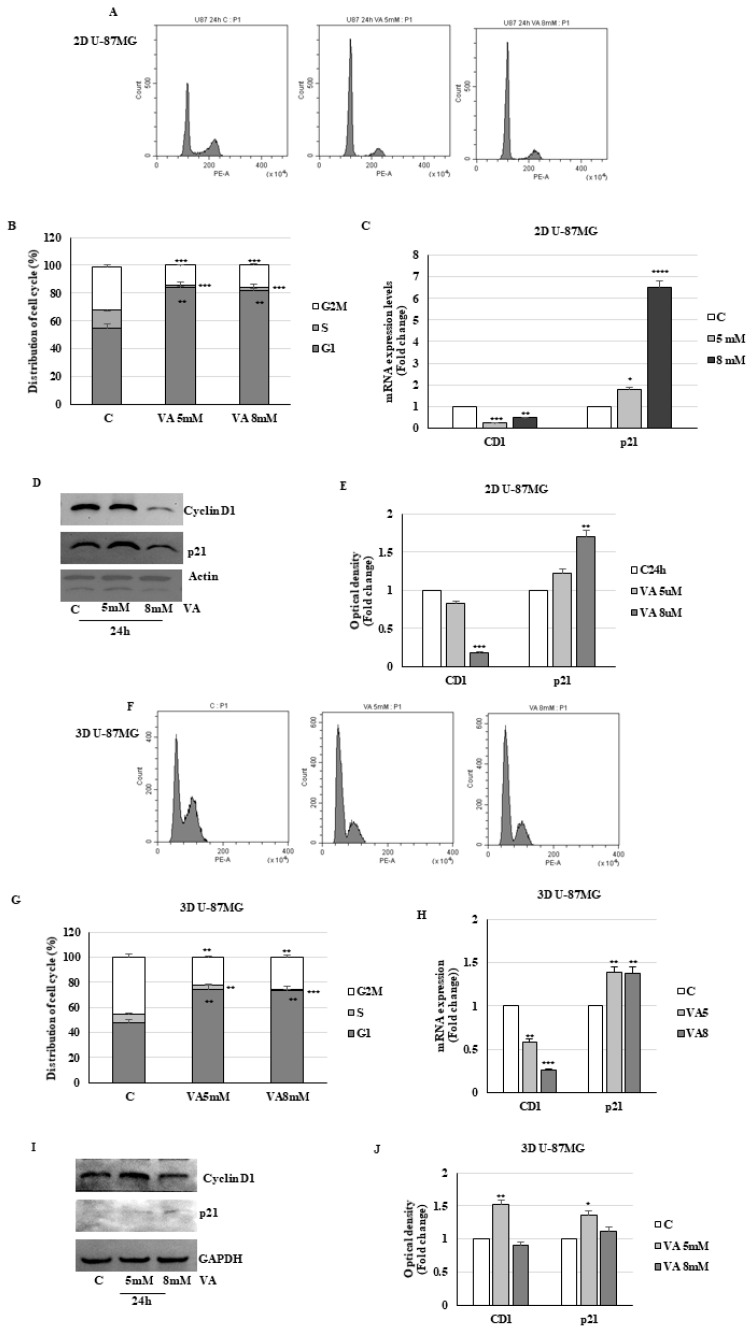
Action of VA on cell cycle inU-87MG glioma cells. Two-dimensional (**A**) and three-dimensional (**F**) U-87MG cells were treated with VA, as indicated, for 24 h, stained with propidium iodide (PI), and analyzed on a flow cytometer. Quantitative analyses of percentage gated cells at G0/G1, S, and G2/M phases are shown (**B**,**G**). The histograms represent the mean ± S.D. of three distinct experiments. ** *p* < 0.01, *** *p* < 0.001. The mRNA levels of cyclin D1 and p21 were evaluated by qRT-PCR using specific primers (**C**,**H**). The 18S mRNA levels were determined to normalize each sample. The histograms represent the mean ± S.D. of three distinct experiments, and the values are represented as fold changes compared to the control set, equal to 1. * *p* < 0.05, ** *p* < 0.01, *** *p* < 0.001, **** *p* < 0.0001 vs. C. Protein levels of cyclin D1 and p21 were analyzed by Western blot (**D**,**I**). The histograms represent the mean ± S.D. of three distinct experiments, and the values are represented as fold changes compared to the control set, equal to 1 (**E**,**J**). * *p* < 0.05, ** *p* < 0.01, *** *p* < 0.001.

To determine whether the cell growth inhibition reported above was a consequence of cell-cycle perturbation, flow cytometry cell cycle analysis was then performed in 2D and 3D T98G and U-87MG cells treated with VA for 24 h. As shown in Figure 7A,B, in monolayer cell cultures of T98G, we observed a significant dose-dependent increase in the percentage of G1 cells from 55.49% of control to 90.3% and 92.6% at 2 and 4 mM VA, respectively, with a reduction in the S phase (control cells 15.16%, VA 2 mM 4.16%, and VA 4 mM 2.04%). Analogously in monolayer U-87MG cells, VA induces a block in the G0/G1 phase, with a marked decrease in the S phase (Figure 8A,B).

The G1 cell-cycle arrest induced by VA in 2D glioblastoma cells was coincident with decreased mRNA and protein levels of cyclin D1, the key regulator of G1 in S phase progression, and increased expression of CDK inhibitor p21 mRNA and protein (Figure 7C–E). VA treatment of U87-MG cells yielded comparable effects on cyclin D1 and p21 mRNA and protein expression levels (Figure 8C–E). Analogously, in drug-treated spheroid cells, we observed an increase in the G1 phase compared to the control group (Figure 7F,G and Figure 8F,G). This data was also consistent with the decrease in the mRNA and protein levels of cyclin D1 in 3D T98G cells. In the same cells, the mRNA of the p21 inhibitor was reduced, while the protein level was increased. This suggests that post-transcriptional mechanisms are involved in the protein stability (Figure 7H–J).

Analogous results were obtained for the 3D U-87MG cell line with respect to the expression of the regulatory proteins p21 and cyclin D1 (Figure 8H–J).

### 2.3. VA Elevates Intracellular ROS Levels in Glioma Cells

Given that most anticancer agents generate intracellular levels of reactive oxygen species (ROS) to induce cell death, we wanted to ascertain whether valproate has an impact on the production of ROS in glioblastoma cells.

**Figure 9:**
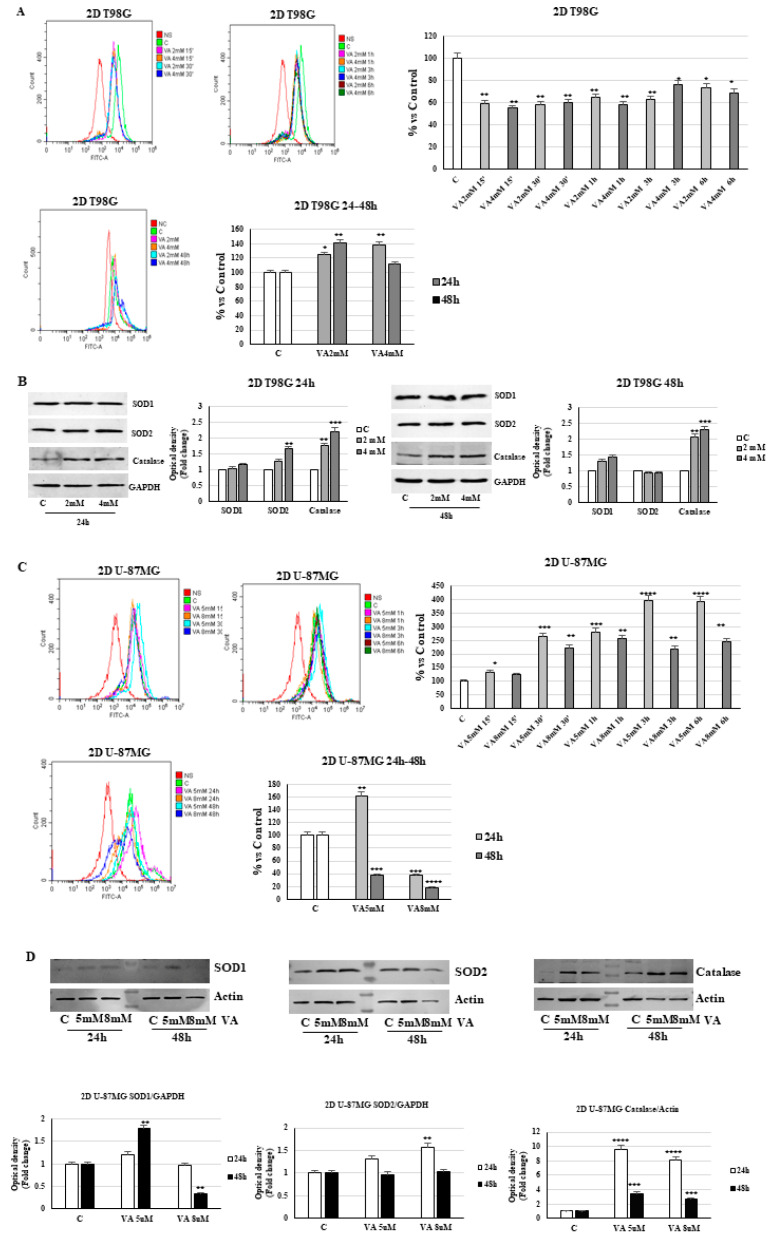
ROS production in VA-treated 2D T98G and U-87MG cells. (**A**) Intracellular ROS levels were analyzed in T98G (**A**) and U-87MG (**C**) cells at different times. Untreated cells (NC), without fluorescent probes, were used as a negative control. Values represent the mean of three triplicate independent experiments. * *p* < 0.05, ** *p* < 0.01 vs. C. Immunoblotting of expression levels of SOD1/2 catalase in glioblastoma T98G (**B**) and U-87MG (**D**) cells. Histograms represent the mean ± SD of three experiments. Band intensities were evaluated as arbitrary units of optical density (OD) and expressed as fold changes relative to control (C). ** *p* < 0.01, *** *p* < 0.001, **** *p* < 0.0001 vs. C.

The generation of intracellular ROS was measured using the fluorescent probe CM-H2DCFDA by flow cytometry. As shown in Figure 9A, a marked decrease in the fluorescent signal was observed in 2D T98G cells during the early treatment period with VA (up to 6 h), whereas the signal increased notably after 24 h of incubation time and, above all, at 4 mM with respect to control. Under the same experimental conditions, Western blot analyses showed a dose-dependent upregulation in the expression levels of the antioxidant proteins superoxide dismutase 1 (SOD1) and catalase for 24 and 48 h of treatment, while the mitochondrial superoxide dismutase 2 (SOD2) increases only at 24 h of treatment (Figure 9B).

In contrast to the T98G cells, the data obtained from the 2D U-87MG cell line showed an early increase (up to 6 h) in ROS production in treated cells, which persists at 24 h with 5 mM VA, while a reduction is observed at 24 and 48 h, particularly with 8 mM VA (Figure 9C). Regarding antioxidant enzymes, SOD1 expression increases in 2D U-87MG cells at 24 and 48 h of treatment with 5 mM VA but decreases at higher valproate concentrations after 48 h. In contrast, SOD2 levels remain higher than in untreated cells only at 24 h of incubation. Valproate, however, maintains elevated catalase levels at both treatment time points (Figure 9D).

ROS detection in spheroids does not always reflect the results obtained in monolayers. In fact, in 3D T98G cells, valproate does not induce ROS production, which remains below control levels (Figure S2). In contrast, the data obtained from U-87MG spheroids are consistent with those observed in the same cells grown as monolayers (Figure S2).

### 2.4. The Intrinsic Apoptosis Pathway Is Activated by VA

Based on the above results concerning the antiproliferative effects and ROS production induced by valproate in glioblastoma cells, we proceeded to explore the potential contribution of the apoptotic response. In order to do so, we examined the expression of pro- and antiapoptotic proteins together with the evaluation of caspase-9 and caspase-3.

**Figure 10:**
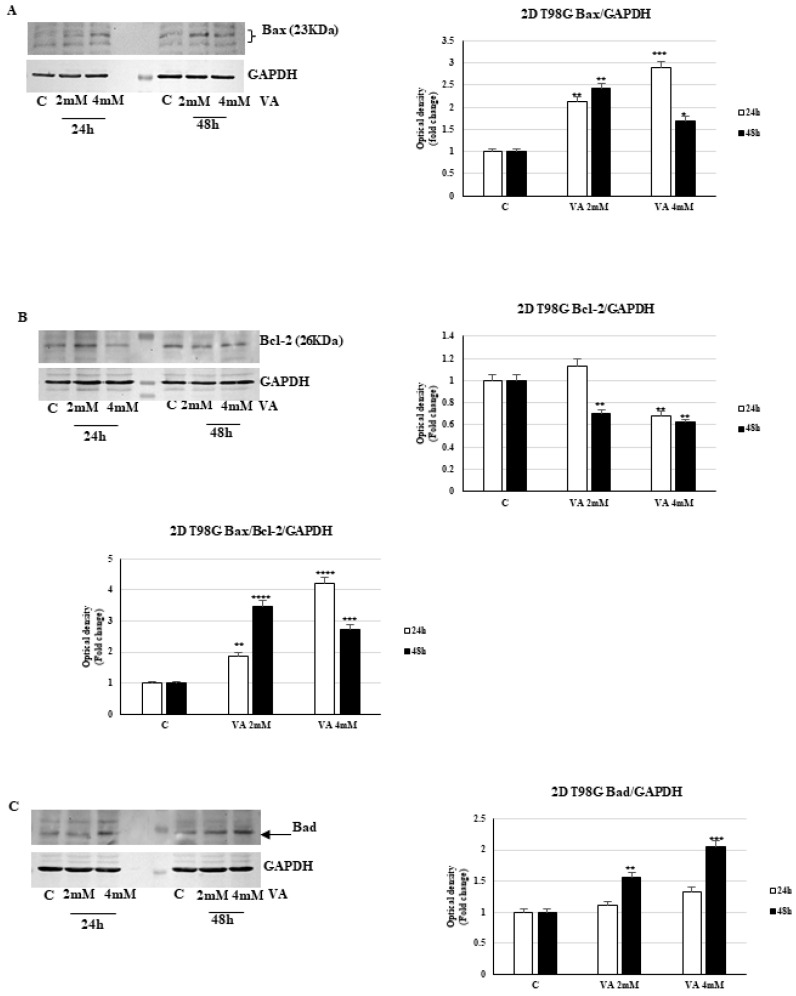
VA addresses apoptosis in glioma cells. Immunoblotting of expression levels of Bax (**A**), Bcl-2 (**B**) and Bad (**C**), in glioblastoma 2D T98G cells. Histograms represent the mean ± SD of three experiments. Band intensities were evaluated as arbitrary units of optical density (OD) and expressed as a fold change relative to control. * *p* < 0.05, ** *p* < 0.01, *** *p* < 0.001, **** *p* < 0.0001 vs. C.

**Figure 11:**
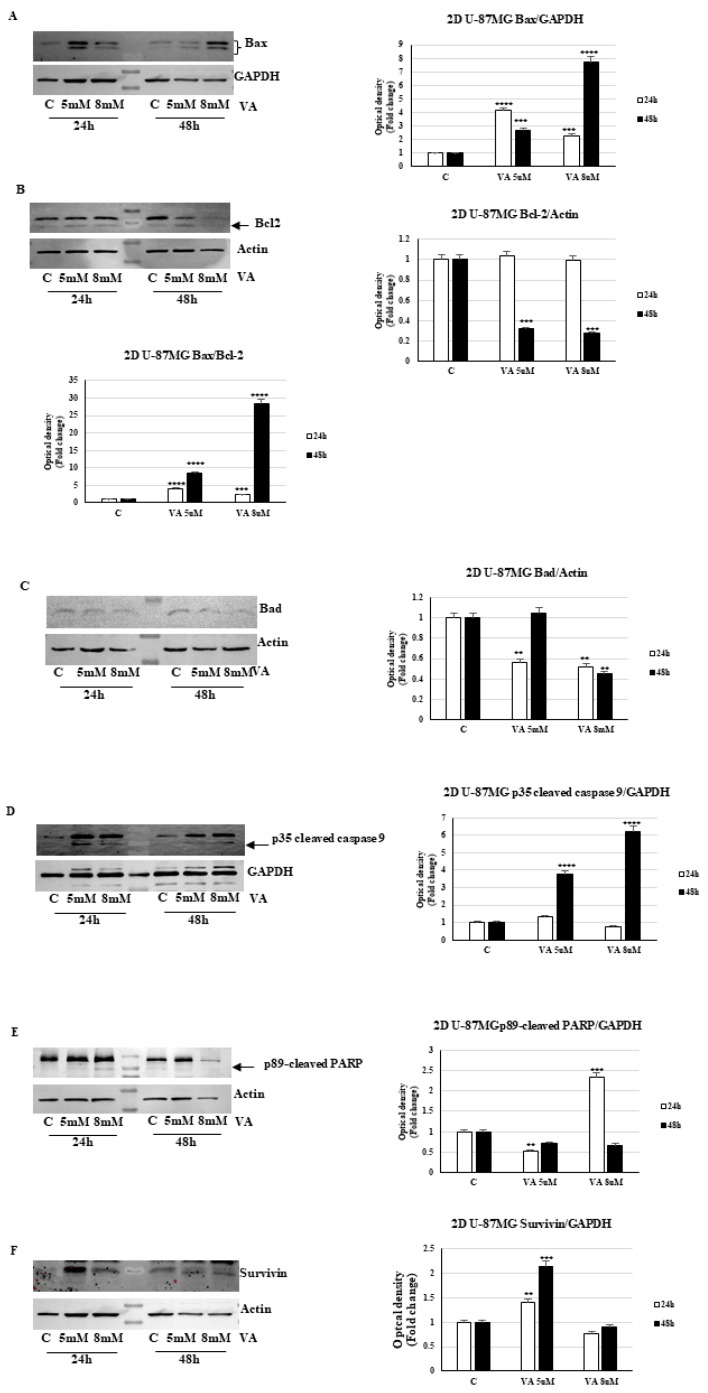
Role of VA on apoptosis-related molecules expression in 2D U-87MG cells. Protein levels of Bax (**A**), Bcl-2 (**B**), Bad (**C**)caspase-9 (**D**), a cleaved fragment of PARP (**E**) and survivin (**F**) in glioblastoma 2D U-87MG cells, analyzed by WB. The histograms represent the mean ± SD of three experiments, in which the band intensities were evaluated in terms of arbitrary units of optical density (OD) and expressed as a fold change relative to the control. ** *p* < 0.01, *** *p* < 0.001, **** *p* < 0.0001 vs. control.

Western blot analysis demonstrated that VA induced apoptosis in T98G and U-87MG monolayer cells by lowering Bcl-2 levels and enhancing the expression of the proapoptotic proteins Bax and Bad, although the latter protein is not increased under VA in U-87MG cells. Furthermore, it has been observed that there is a significant increase in the Bax/Bcl-2 ratio after 48 h of treatment compared to control cells (Figure 10 and Figure 11).

**Figure 12:**
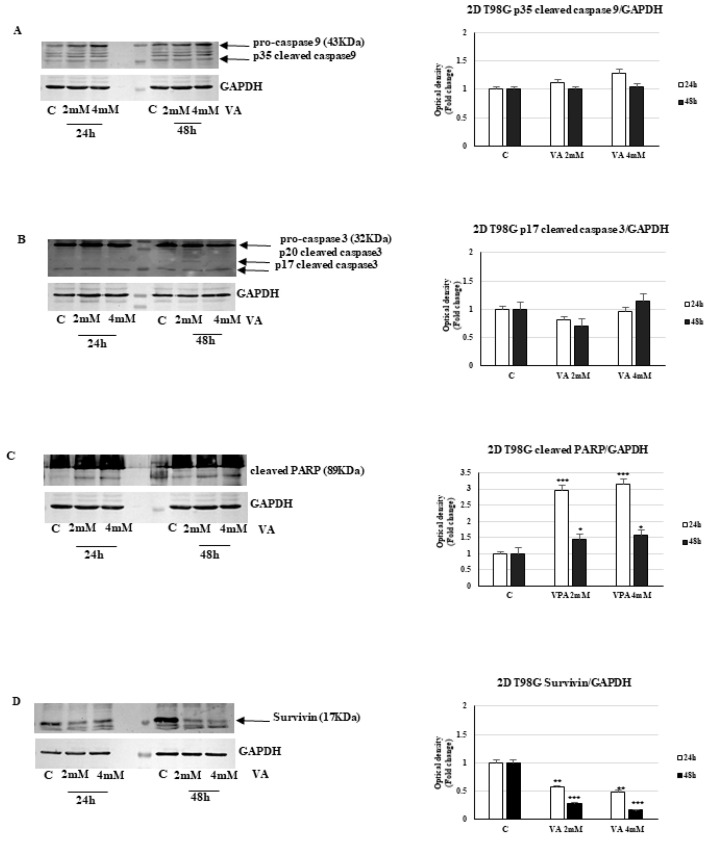
Role of VA on apoptosis-related molecules expression. Protein levels of caspase-9 (**A**), caspase-3 (**B**), a cleaved fragment of PARP (**C**), and surviving (**D**) in glioblastoma 2D T98G cells, analyzed by WB. The histograms represent the mean ± SD of three experiments in which the band intensities were evaluated in terms of arbitrary units of optical density (OD) and expressed as a fold change relative to the control. * *p* < 0.05, ** *p* < 0.01, *** *p* < 0.001 vs. control.

Under the same experimental conditions, in 2D T98G cells, we observed the cleaved forms of initiator caspase-9 (37 kDa) and executioner caspase-3 (20 and 17 kDa) at both concentrations of VA (Figure 12). Once activated, caspase-3 can cleave several substrates, one of which is PARP (poly ADP ribose polymerase). Indeed, VA-treated T98G cells exhibited a significant increase in the proteolytic form of PARP (89 kDa) compared to controls at both drug concentrations (Figure 12). On the contrary, a reduction in the expression of the small inhibitory protein survivin, which belongs to the IAP (Inhibitors of Apoptosis Proteins) family, was revealed in cells that underwent VA treatment (Figure 12). This protein plays a crucial role in regulating apoptosis, but also in tumorigenesis. Indeed, its overexpression in various tumor types is associated with an unfavorable prognosis as it contributes to resistance against the apoptotic effects of many chemotherapeutic agents [24].

As for caspase signaling, the data from U-87MG cells only partially align with those obtained in the T98G cell line. U-87MG cells exhibit a weaker response to valproate with respect to these markers. In particular, only caspase-9 shows cleavage, while caspase-3 could not be detected at all as no bands were observed in the Western blot. PARP cleavage is evident in U-87MG cells only at 48 h with 8 mM VA; after this time point, cleavage is no longer detectable, and PARP levels return to those of the control. Moreover, surviving levels do not decrease but instead remain comparable to, or even higher than, control levels (Figure 11).

**Figure 13:**
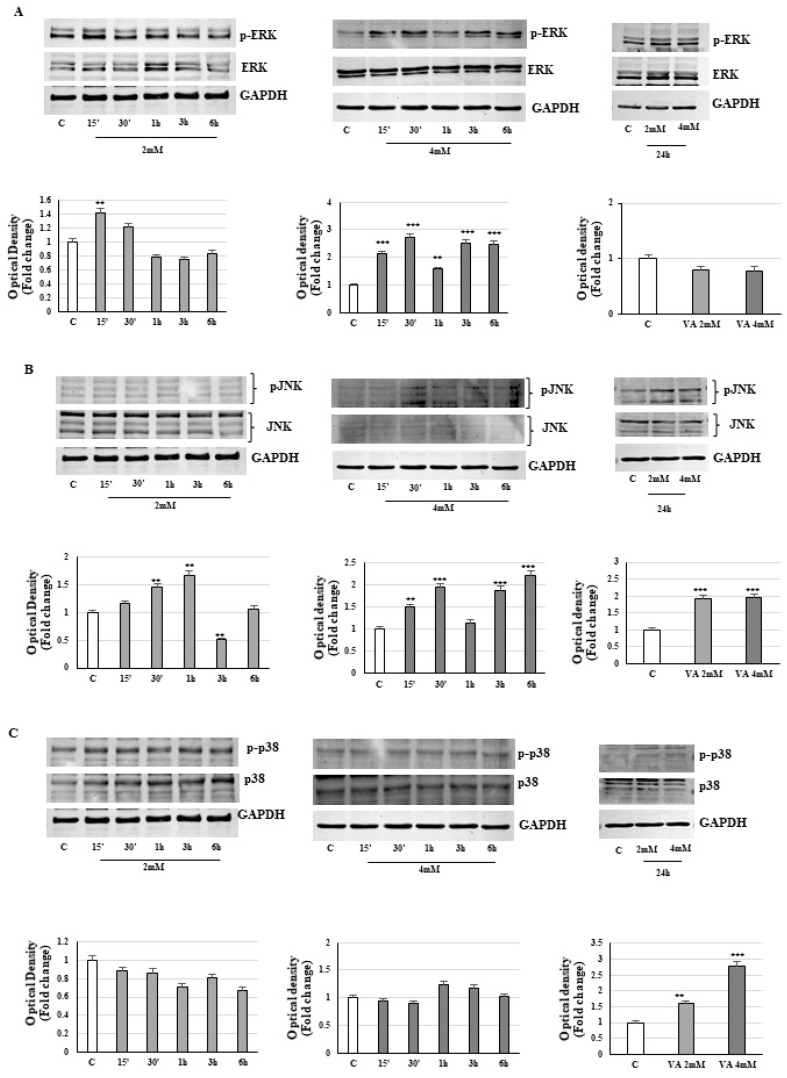
VA influences the MAPK pathways in 2D T98G. Immunoblotting of phospho-ERK (**A**)/JNK (**B**)/p38 (**C**) and relative protein levels. The histograms represent the mean ± SD of three experiments, in which the band intensities were evaluated in terms of arbitrary units of optical density (OD) and expressed as a fold change relative to the control. ** *p* < 0.01, *** *p* < 0.001 vs. control.

Apoptosis and several pathophysiological processes during stress may be linked to the activation of MAPK family members, including p38, the JNK, and ERK1/2, that affect the cell’s fate in a different way [25]. Considering that phosphorylation events are rather immediate, MAPK signaling in 2D glioblastoma-treated cells was evaluated at short time points and after 24 h. The data presented in Figure 13A show that valproic acid induces an early and transient activation of ERK1/2. However, over a longer period of time, a sustained activation of stress signals, such as p38 and JNK, was observed (Figure 13B,C).

The MAPK signaling pattern analyzed in T98G spheroid cells, revealed a valproate-induced activation of ERK1/2, JNK, and p38 at 24 h, suggesting an enhanced stress response in the 3D system (Figure S3).

## 3. Discussion

Glioma, the most common type of primary brain cancer, accounts for more than 70% of central nervous system tumors [26].

Glioblastoma has a high incidence, recurrence rate, and mortality, with limited treatment options. The main approaches include surgical resection, radiotherapy, and chemotherapy with the alkylating agent temozolomide [27].

Although new therapeutic strategies have been introduced in recent years, such as the use of antiangiogenic agents like bevacizumab or new chemotherapy protocols, GBM remains incurable due to its phenotypic, morphological, and cellular heterogeneity, as well as its pathological complexity.

Epigenetic drugs such as HDACi are emerging as potential future antitumor therapies [11,28].

In particular, valproic acid appears to be one of the most promising HDACi, showing strong antitumor activity in a wide range of cancer cells, including GBM, although its exact mechanism of action is still not fully understood, involving multiple biological processes and signaling pathways [29,30].

In breast and prostate cancer cell lines, valproic acid inhibited cell proliferation, blocked the cell cycle, and activated apoptosis [17,18,19]. In other tumor cell types, it was mainly implemented as an adjuvant drug to chemotherapy, radiotherapy, and further treatments [20,31].

The first experimental findings of our study showed that VA inhibits the viability of T98G and U-87MG cells and induces cell cycle arrest in the G1 phase, along with the downregulation of cyclin D1 expression. In general, the cyclin family activates cyclin-dependent kinases (CDKs), which drive cell cycle progression by forming active complexes with CDK4 and CDK6, leading to phosphorylation of the retinoblastoma protein (pRb) and promoting the G1-to-S phase transition [32]. This activity is counteracted by p21, which binds to cyclin D1–CDK4/6 complexes and inhibits their kinase function. Therefore, the balance between cyclin D1 and p21 expression plays a pivotal role in determining whether the cell progresses through the cycle or undergoes arrest. In line with this regulatory mechanism, the valproate-induced upregulation of the p21^Cip1/WAF1^ protein, a member of the Cip/Kip family of CDK inhibitors in 2D cultures, may underlie the molecular basis of the observed proliferation arrest [33].

In accordance with these data, the upregulation of the p21^Cip/WAF1^ protein, a member of the Cip/Kip inhibitor family, induced by VA in the 2D cells, elucidates the molecular mechanism through which the proliferative block occurs. Furthermore, valproate is able to counteract 2D T98G cell migration, as demonstrated by the wound healing assay, showing a significant effect after just 9 h of treatment at the highest VA concentration. In addition, the reduction in mRNA and the activation protein levels of MMP-2 confirm that valproate effectively modulates the invasive potential of 2DT98G cells. It is well known that in transformed phenotypes, MMP-2 enables tumor invasion and angiogenesis via the degradation of ECM components like collagen, fibronectin, laminin, and elastin. Additionally, MMP-2 facilitates the reorganization of the extracellular matrix, a process essential for the growth and branching of dendrites in nerve cells. This is crucial for the formation of synaptic connections during nervous system development. In glioma, it has been reported that MMP-2 expression correlates with high pathological grade and increased invasiveness, thus representing a negative prognostic factor [31,34].

The increase in motility and the acquisition of the metastatic phenotype in tumor cells are processes that involve the epithelial–mesenchymal transition (EMT).

The role of EMT in glioma progression is not fully understood, and it has been referred to as an EMT-like process [5]. However, is known to contribute to tumor progression, chemoresistance, and recurrence after treatment. In particular, GBM cells are not typical epithelial cells; they do not have a basement membrane in the neuronal environment and do not consistently express E-cadherin [35]. Elevated expression of classic EMT markers can transform GBM cells into a more invasive mesenchymal subtype in the neuroepithelial milieu, contributing to a worse prognosis and resistance to treatment [36].

In neural tissue, N-cadherin plays a critical role during neurulation, forming adherens junctions to maintain neural tissue architecture and regulating neural progenitor proliferation and differentiation [37]. Observations in glioma samples suggest that N-cadherin expression varies and that this protein drives the polarization and migration capacity of astrocytes. Reduced expression of N-cadherin is typically found in high-grade gliomas compared to normal primary glial cell cultures. Therefore, N-cadherin levels influence, at least partly, the potential motility and spread of gliomas [38,39].

Nonetheless, variations in N-cadherin expression are still disputed due to differences between glioma samples, tissue-derived glioma cells, and glioma cell lines. Overexpression of vimentin, alpha-smooth muscle actin (α-SMA), and twist, which are biomarkers of the mesenchymal phenotype, have been correlated with glioma progression and poor prognosis [5].

Hence, based on the information provided, efforts to counteract EMT in cancers and to restore epithelial characteristics have progressed over the years. In this regard, the use of small molecules of different origins, including micro-RNA, has been shown to reverse the EMT phenotype and overcome cancer drug resistance [5,40]. The effects of valproic acid on EMT-related gene expression in glioblastoma cell lines appear to be highly context-dependent, varying with both cell line identity and culture dimensionality.

Our findings demonstrated that valproate treatment in glioblastoma T98G cells effectively reduced the mRNA levels of vimentin, alpha-SMA, and twist, while simultaneously increasing the expression of the epithelial protein E-cadherin. On the other hand, the sustained expression of N-cadherin in VA-treated GBM cells support previous reports showing that decreased levels of N-cadherin promote glioma migration and invasiveness [39].

The 2D U-87MG cells exhibited a clear shift toward a mesenchymal phenotype following VA treatment, with the upregulation of N-cadherin, vimentin, and α-SMA, alongside a reduction in E-cadherin expression. This pattern suggests that in monolayer culture, VA may potentially promote EMT by relieving the transcriptional repression of EMT-associated transcription factors.

In the scope of our study, we included 3D cultures of glioblastoma cells as they more accurately replicate the biological behavior of a tumor.

In fact, the 3D cellular model provides more physiologically accurate and realistic interactions between cells and their milieu. Cancer cells may spread more heterogeneously, acquire various phenotypes, and gain metastatic potential and drug resistance that resemble those of human tumors [41]. A 3D spheroid model of a GBM, in vitro, is composed of continuously dividing cells, quiescent cells, and necrotic cells, which are the three main cell types present in the tumor represented as centric layers in the anatomy of the tumor mass [42].

The results presented here highlight how valproate effectively reduces cell mass growth and arrests cells in the G1 phase of the cycle, consistent with the lowering of cyclin D1. The 3D glioblastoma models showed enhanced sensitivity to VA compared to the 2D culture, particularly in reducing the mRNA levels of the mesenchymal markers investigated. In 3D cultures, U-87MG spheroids treated with VA showed a reversal of the EMT profile observed in 2D, characterized by decreased expression of mesenchymal markers and a significant upregulation of E-cadherin. This suggests that in a 3D microenvironment, where cells are exposed to more physiological cues such as hypoxia, mechanical stress, and cell–matrix interactions, VA may instead inhibit EMT, potentially favoring a less invasive, more differentiated epithelial-like state. Unlike what was observed in monolayer cell cultures, N-cadherins were decreased by VA in spheroid cells. This is in line with previous studies on glioblastoma cells, where a different expression of the proteins involved in intercellular junctions has been reported in 2D and 3D cellular models [43].

Interestingly, the broad downregulation of both mesenchymal markers and E-cadherin in 3D T98G cultures might indicate a possible transition of the cells to a quiescent or stress-adaptive state.

Next, our study demonstrated that VA treatment in the 2D glioblastoma cell line induces an overproduction of reactive oxygen species (ROS). The role of ROS in tumorigenesis is controversial as they have been shown to possess both tumor-promoting and tumor-inhibiting properties [44]. According to the scientific literature, a threshold exists for intracellular ROS concentrations. Specifically, low-to-moderate levels of ROS can be advantageous for cells, stimulating growth processes and DNA mutations, while elevated ROS levels can be detrimental and lead to cell death. In glioma cells, it has been shown that ROS accumulation results in cell arrest and death, whereas a decrease in ROS boosts cell survival [45]. In cultured glioma cells and other cancer cells, salinomycin, an antibacterial drug, alters mitochondrial membrane permeability and induces ROS over-production, which is crucial for address cell necrosis [46].

Given that elevated ROS can have cytotoxic effects, anticancer agents that induce oxidative stress represent a promising therapeutic strategy [46,47,48,49]. In 2D T98G cells, we did not observe any significant increase in ROS levels during the early intervals. While the effect became apparent only after prolonged exposure, particularly at 24 and 48 h, this increase in ROS was accompanied by a concomitant upregulation of antioxidant enzymes, including SOD1, SOD2, and catalase, which are typically activated as part of the cellular defense mechanism to counteract oxidative stress. This suggest that in T98G cells, ROS generation is not an early event and may instead result from later processes such as cell death. In contrast, 2D U-87MG cells show an early increase in ROS levels starting from 15 min post-treatment. This supports the conclusion that ROS induction is a primary event and likely contributes to the initiation of cell death, rather than it being a consequence of thereof.

Meanwhile, further evidence from different cancer cell lines indicates that ROS generation may play a role in triggering apoptosis and activating stress-related MAPKs [50,51,52].

Here, it has been proven that VA triggers cell apoptosis as result of increased levels of the proapoptotic proteins Bax and Bad, along an elevated proapoptotic/antiapoptotic (Bax/Bcl-2) ratio. Accordingly, VA triggers cell apoptosis through the cleavage of caspases and PARP.

On the other hand, a significant reduction in the protein survivin, which belongs to the IAP (inhibitors of apoptosis proteins) family, was observed after treatment of monolayer T98G cells.

Survivin has been shown to suppress apoptosis and regulate cell division by binding to caspase-9 and to the downstream effector caspase-3 and caspase-7, thus inhibiting the apoptosis process. Inhibitors of IAPs in glioblastoma sensitize cells to TRAIL-induced apoptosis and to radiation [53,54].

Analogously, the dietary phytochemical piperine was able to inhibit survivin in glioblastoma, thus affecting stemness, cancer progression, and the resistance of the cells [55].

After exposing monolayer T98G glioblastoma cells to valproic acid, we noticed a temporal activation of MAPK p38 and JNK phosphorylation, coinciding with the proliferative halt, the ROS production and the apoptotic response. Instead, phosphorylation of p42/44 MAPK transiently appears during short treatment times, while with longer exposure periods, it tends to fall below control levels. Mitogen-activated protein kinases (MAPKs), a family of serine/threonine kinases, have been implicated in the balance between cell survival and death in response to stress signals.

In particular, p38 and JNK mitogen-activated protein kinases have mentioned as mediators of stress and inflammation responses in different cellular types [56,57].

Many chemotherapeutic agents induce an apoptotic response through the activation of both MAPK pathways, which also increase chemosensitivity to drug treatment [58,59].

However, depending on certain conditions, p38 MAPK can also mediate resistance to apoptosis, as it occurs in prostate, oral, and lung cancer cells [60,61,62].

It is noteworthy that the JNK and p38MAPK signaling pathways might either promote cancer or suppress tumor cellular growth, with underlying mechanisms that require further exploration in the cellular context [63].

Nevertheless, studies on glioblastoma cells have emphasized that the natural alkaloid piperlongumine (PL) and the steroid saponin PPI function as anticancer agents, being able to induce cell death by activating the JNK and p38 MAPK signaling pathways [64,65].

In our context, the results confirm that in 2D glioma cells, the above-mentioned kinases are activated by the VA treatment and are involved in the antitumoral action of the drug. Our data suggest that ERK1/2 activation is not significantly altered by VA treatment, supporting the idea that this pathway may not play a central role in the observed cellular response.

In contrast, the marked increase in phosphorylated JNK and p38 levels after 24 h could indeed reflect a stress response associated with ongoing cell death processes; thus, JNK and p38 activation might be a downstream consequence rather than a direct mediator of VA-induced effects.

Beyond its role as an antiepileptic, valproate is effective in many types of tumor cell lines since it inhibits cell proliferation and survival by addressing apoptosis. Additionally, valproic acid has been investigated for its potential neuroprotective role under the same neurodegenerative conditions. Evidence shows that the drug enhances neuronal survival, lowers neuroinflammation, and improves cognitive abilities in animal models [66]. These properties grant the application of the drug its status as a health-promoting pharmacological approach.

In conclusion, our study indicates that VA can antagonize various cellular processes, such as the viability and migration of glioma cells, while promoting cell apoptosis. However, these effects are not uniform across the two models.

Furthermore, the comparative analysis between the 2D and 3D cell models was crucial as it clearly showed that valproic acid, in both experimental setups, can significantly reduce glioblastoma cell viability in 2D models, reduce the cellular mass of spheroids, inhibit EMT, and hinder cancer cell migration.

Future research will aim to investigate the action of VA, in combination with other compounds, on molecular mechanisms that control the survival, invasiveness, and stemness in 3D glioblastoma experimental models, with the goal of uncovering new diagnostic markers to be explored for therapeutic outcomes.

## 4. Materials and Methods

### 4.1. Cell Culture

Human glioblastoma cell line T98G and U-87MG, as well as SVGp12 cells, a human fetal glial cell line derived from normal human astroglial cells, were purchased from the American Type Culture Collection (ATCC, Manassas, VA, USA). Cells were stored according to supplier’s instructions and regularly tested for mycoplasma negativity (MycoAlert Mycoplasma Detection Assay, Lonza, Basilea, Switzerland). T98G, U-87MG, and SVG p12 cells were cultured in Minimum Essential Medium (Life Technologies, Monza, Italy) supplemented with 10% fetal bovine serum (FBS), 200 mM L-glutamine, 1% penicillin–streptomycin, 1% Eagle’s nonessential amino acids, and 1% sodium pyruvate (Life Technologies, Monza, Italy) at 37 °C in a humidified atmosphere with 5% carbon dioxide and 95% air.

### 4.2. Cell Viability Assay

Cell viability was evaluated using 3-[4,5-dimethylthiazole-2-yl]-2,5-diphenyltetrazolium bromide (MTT)-based colorimetric assay (Sigma-Aldrich, Merck, Milan, Italy). T98G, U-87MG and SVG p12 cells were seeded in 96-well cell culture plates (Tissue Culture Treated, Corning, Sial, Italy) at a density of 5 × 10^3^ cells/mL and were grown in complete media at 37°C and 5% CO_2_ in HERA cell 150 (ThermoFisher Scientific, Milan, Italy) incubator. After the attachment, cells were starved with phenol red-free and serum-free media for 24 h and then untreated (control, C) or treated with VA at different concentrations (1, 2, 4, 6, 8, 10 mM) for 24, 48, and 72 h for human glioblastoma cells, while SVG p12 were treated at concentrations of 1, 2, 4, 5, and 8 mM for 24 and 48 h. At the desired endpoint, the growth medium was removed, and cells were incubated with 100 µL/well of 2 mg/ml of MTT solution for 4 h at 37 °C. Subsequently, MTT solution was replaced with DMSO to dissolve the formed formazan crystals. The absorbance of formazan crystal products was measured by spectrophotometry at 570 nm using a microplate reader (Multiskan EX, ThermoFisher System, Milan, Italy).

### 4.3. Colony Formation Assay

T98G and U-87MG cells were seeded in 6-well cell culture plates (Corning, Sial, Italy) at a density of 3 × 10^3^ cells/well and then untreated (control, C) or treated with VA 1 mM, 2 mM, and 4 mM or VA 1 mM, 5 mM, and 8 mM, respectively, for the cell type. Treatments were renewed every 2 days. After 14 days, cells were gently washed with PBS, fixed in acetone–methanol (1:1) for 5′ at room temperature, and then stained with 0.1% Crystal Violet solution for 30′. The colonies formed were photographed using a digital camera and counted by ImageJ program.

### 4.4. Soft Agar Anchorage-Independent Growth Assay

In order to evaluate anchorage-independent cell growth, soft agar colony formation assay was performed. T98G and U-87MG cells (3 × 10^3^/well) were plated in 2 mL of 0.35% agarose in phenol red-free media on top of a 1% of agarose base layer in 6-well cell culture plates (Corning, Sial, Italy). One day after plating, growth media containing VA 1 mM, 2 mM, and 4 mM or VA 1 mM, 5 mM, and 8 mM, respectively, for the cell type, was added to the top layer and replaced every two days. After 14 days, 300 μL of MTT (Sigma-Aldrich, Merck, Milan, Italy) were added to each well and incubated for 4 h at 37 °C. Plates were then placed overnight at 4 °C, and colonies >50 μm in diameter were counted using a phase contrast microscope.

### 4.5. Wound Healing Assay

T98G cells were seeded at a density of 1.5 × 10^5^ cells/well in 6-well Cell Culture plates (Corning, Sial, Italy). Once 100% confluence was reached, a scratch (wound) was made using a 10 μL pipette tip. Cellular debris was gently removed by washing with DPBS, and subsequently, cells were cultured in the absence or presence of VA. Randomly selected fields were immediately captured (at 0 h) using a phase-contrast microscope at 4× magnification. The images were taken using a sCMEX-3 microscope camera (Euromex) and Image Focus Alpha software (version x64, 1.3.7.26221.20240804). Then, cells were maintained at 37 °C and 5% CO_2_, and images were captured at identical locations at 9, 18, and 24 h. The wounding area was measured and analyzed using ImageJ Software, and the wounding closure rate was established as follows:

{[(wound area at time 0) − (wound area at time x)/(wound area at time 0)] × 100}

### 4.6. Detection of MMP-2 Activity by Zymography

The T98G cells were treated or untreated with VA at final concentrations of 2 and 4 mM for 24 h. At the end of the treatment, cells were collected, and total protein extracts were quantified using the Bradford method to ensure equal protein loading before being prepared for zymographic analysis. Briefly, equal amounts of protein (60 μg) were precipitated with ice-cold acetone at −20 °C for 1 h and centrifuged, and the resulting pellets were resuspended in loading buffer [125 mM Tris-HCl (pH 6.8), 4% SDS, 10% glycerol, 0.10% bromophenol blue]. Samples were then loaded and separated under non-denaturing conditions on 8% SDS-PAGE gels containing 0.1% (*w*/*v*) gelatin. After electrophoresis, gels were washed for 30 min at room temperature in renaturation buffer [50 mM Tris-HCl (pH 7.5), 10 mM CaCl_2_, 2.5% Triton X-100] to remove SDS and allow for enzyme renaturation. Gels were then incubated overnight at 37 °C in activation buffer [50 mM Tris-HCl (pH 7.5), 10 mM CaCl_2_, 1% Triton X-100] to enable enzymatic activity. Finally, gels were stained with 0.2% (*w*/*v*) Coomassie Brilliant Blue R-250 (Sigma Aldrich, St. Louis, MO, USA) in 45% methanol and 10% acetic acid, followed by destaining in 10% methanol and 10% acetic acid until clear bands appeared. Gelatin digestion areas, indicative of MMP activity, were visualized and quantified using ImageJ 1.6 software. MMP-2 activity was expressed as a percentage of the total band area across the three experimental conditions.

### 4.7. Cell Cycle Analysis

Flow cytometry analysis was performed to evaluate the percentage of T98G and U-87MG cells, cultured in 2D and 3D conditions, in the different phases of the cell cycle. For 2D cultures, cells were seeded in 6-well cell culture plates (Corning, Sial, Italy), synchronized in phenol red-free and serum-free medium for 24 h, and subsequently left untreated (control, C) or treated with VA at 2 mM and 4 mM for T98G, or 5 mM and 8 mM for U-87MG, for 24 h. For 3D cultures, T98G and U-87MG cells were plated as single-cell suspensions (3 × 10^5^ cells per well) in ultra-low attachment 6-well plates (Corning, Sial, Italy), and were either left untreated (control, C) or treated with the same concentrations of VA. To generate three-dimensional spheroids, the plates were rotated for 4 h at 37 °C. After the treatment, cells obtained from monolayer cultures and disaggregated spheroids were pelleted, washed with PBS, and fixed in 50% methanol overnight (ON) at −20 °C. Then, cells were stained with a solution containing 50 μg/ml propidium iodide (PI), 20 U/ml RNase, and 0.1% Triton. Cell cycle phases were estimated as a percentage of a total of 10.000 events. The DNA content was measured using CytoFLEX flow cytometry (CytoFLEX Beckman, Beckman-Coulter, Milan, Italy), and the data were acquired using CytExpert Beckman Coulter software (version 2.4).

### 4.8. Real-Time PCR

Total RNA was isolated from U87-MG and T98G cells, cultured in 2D or in 3D, and untreated (control, C) or treated with VA for 24 h using TRI-zol reagent (ThermoFisher Scientific, Milan, Italy). cDNA was generated by reverse transcription (2 μg) with the High-Capacity cDNA Reverse Transcription Kit (ThermoFisher Scientific, Milan, Italy) according to the manufacturer’s instructions and was diluted 1:10 in nuclease-free water. Gene expression was assessed by real-time reverse transcription PCR (qRT-PCR) with QuantStudio™ 3 Real-Time PCR System (ThermoFisher Scientific, Milan, Italy) using SYBR Green Universal PCR Master Mix (Bio-Rad, Milan, Italy). Each sample was normalized on its 18S mRNA content. Relative gene expression levels of the amplification products were calculated and analyzed using the 2^−ΔΔCq^ method as previously described [67]. Primers used are listed in Table 1.

### 4.9. Immunoblotting Analysis

T98G and U-87MG cells, grown in 2D and 3D conditions and untreated (control, C) or treated with VA 2 mM and 4 mM or VA 5 mM and 8 mM, respectively, for 24 h, were lysed with ice-cold RIPA buffer containing 50 mM HEPES (pH 7.5), 150 mM NaCl, 1.5 mM MgCl2, 1 mM EGTA, 10% glycerol, and 1% Triton X-100, and supplemented with protease inhibitors (1.7 mg/mL aprotinin, 1 mg/mL leupeptin, 200 mmol/L phenylmethylsulfonyl fluoride, 200 mmol/L sodium orthovanadate and 100 mmol/L sodium fluoride) (Sigma-Aldrich, Merck, Milan, Italy). The protein content was determined using Bradford dye reagent (Bio-Rad, Milan, Italy). Equal amounts of total protein were separated to polyacrylamide gel electrophoresis and transferred on to a nitrocellulose membrane by electro blotting. Membranes were blocked with 5% bovine serum albumin (BSA, A9418, Sigma-Aldrich) in Tris-buffered saline-Tween (TBST: 150 mM NaCl, 50 mM Tris-HCl (pH 7.6), 0.1% Tween) with constant agitation at room temperature for 1 h and then incubated with a primary IgG antibody against the following proteins: p-JNK, JNK, p-p38, and p-38 (Invitrogen, Thermo Fisher Scientific); cyclin D1, p21, Bax, Bcl2, Bad, caspase-9, caspase-3, ERK2, survivin, PARP, and GAPDH (Santa Cruz Biotechnology, DBA, Milan, Italy); and p-ERK (Cell Signaling Technology, Euroclone, Milan, Italy). At the end of incubation, the membranes were washed and incubated with an alkaline HRP conjugated secondary IgG antibody. The antigen–antibody complex was revealed by HRP/H_2_O_2_-catalyzed oxidation of luminol in alkaline conditions using an enhanced chemiluminescence system (ECL, Santa Cruz Biotechnology, Milan, Italy). In all immunoblotting experiments, GAPDH and actin were used as a control for the loading of proteins. The immunoreactive bands were detected by chemiluminescence exposing blots to Invitrogen iBright^TM^ Imager 1500 (Thermo Fisher Scientific).

Fiji (image processing package of ImageJ2, v2.16.0) software (version 2.9.0/1.53t) was used to perform the densitometric analysis of blot images.

### 4.10. Reactive Oxygen Species (ROS) Assessment

ROS levels were quantified by flow cytometry (CytoFLEX Beckman, Beckman Coulter, Milan, Italy) in cells stained with a chloromethyl derivative of 2′,7′-dichlorodihydrofluorescein diacetate CM-H2DCFDA (ThermoFisher Scientific, Milan, Italy), a cell-permeable probe that is non-fluorescent until it detects oxidation within the cell. After 24 h of treatment with VA, T98G and U-87MG cells derived from 2D and 3D cell cultures were collected and incubated with 10 μM CM-H2DCFDA (diluted in DPBS) at 37 °C for 30–40 min. Next, the stained cells were harvested by centrifugation and maintained in a fresh medium at 37 °C for 20 min before the flow cytometry analysis. Data were analyzed using CytExpert Beckman Coulter 2.4 software.

### 4.11. Three-Dimensional Cell Culture on Matrigel

T98G and U-87MG cells were plated and treated with VA in MEM for 24 h. Cells were harvested by trypsinization and counted using trypan blue with an automatic cell counter (Countess II Life technologies, ThermoFisher Scientific, Milan, Italy). The 20 µL drops of the cell suspension were pipetted onto the inner side of a sterile 100 mm Petri dish lid (Corning, Sial, Italy). The bottom of the plates was filled with PBS to avoid evaporation. The lid was carefully inverted and placed over the bottom of the Petri dish. The dish was incubated at 37 °C in a humidified atmosphere with 5% CO_2_ for 48 h, allowing the cells to aggregate and form spheroids. After the incubation period, spheroids were collected and resuspended in Matrigel and Collagen mix (1:1) and transferred to 24-well plates containing Matrigel in the bottom. After solidification at 37 °C for 30 min, fresh medium with treatments were added carefully on top of the spheroids, avoiding disruption of the spheroid structures.

Spheroid morphology and size were monitored over time using an inverted phase-contrast microscope (Olympus CKX41, Segrate (MI), Italy). Images were captured, and invasive area was measured using ImageJ analysis software

### 4.12. Statistical Analysis

The results obtained represent the mean ± standard deviation (SD) of at least three independent experiments. Data were analyzed by Student’s *t*-test using the Prism 7.0 (GraphPad Software, San Diego, CA, USA) software program.

## Figures and Tables

**Table 1:** List of primers used.

Oligo Name	Sequence 5′-3′
ALPHA-SMA	Fw-AGACATCAGGGGGTGATGGT
	Rw-CATGGCTGGGACATTGAAAG
CYCLIN D1	Fw-GATGCCAACCTCCTCAACGAC
	Rw-CTCCTCGCACTTCTGTTCCTC
E-CADHERIN	Fw-TGCCCAGAAAATGAAAAAGG
	Rw-GTGTATGTGGCAATGCGTTC
p21	Fw-GCATGACAGATTTCTACCACTCC
	Rw-AAGATGTAGAGCGGGCCTTT
SNAIL	Fw-CGAGTGGTTCTTCTGCGCTA
	Rw-GGGCTGCTGGAAGGTAAACT
TWIST	Fw-TCCAAATTCAAAGAAACAGGGCG
	Rw-CAGAATGCAGAGGTGTGAGGA
VIMENTIN	Fw-GAGAACTTTGCCGTTGAAGC
	Rw-GCTTCCTGTAGGTGGCAATC
18S	Fw-CGGCGACGACCCATTCGAAC
	Rw-GAATCGAACCCTGATTCCCCGTC
N-CADHERIN	Fw-ACAGTGGCCACCTACAAAGG
	Rw-CCGAGATGGGGTTGATAATG

## Data Availability

No new data were created or analyzed in this study. Data sharing is not applicable to this article.

## Supplementary Materials

The following supporting information can be downloaded at: https://www.mdpi.com/article/10.3390/ijms26146600/s1.
