# Supplementary material for: Valproic Acid Reduces Invasiveness and Cellular Growth in 2D and 3D Glioblastoma Cell Lines
Source: Int J Mol Sci. 2025 Jul 9;26(14):6600. doi: 10.3390/ijms26146600 (PMC12294272; doi:10.3390/ijms26146600)
Supplement: Supplementary file 1 [file ijms-26-06600-s001.zip › ijms-3620442-supplementary.pdf]

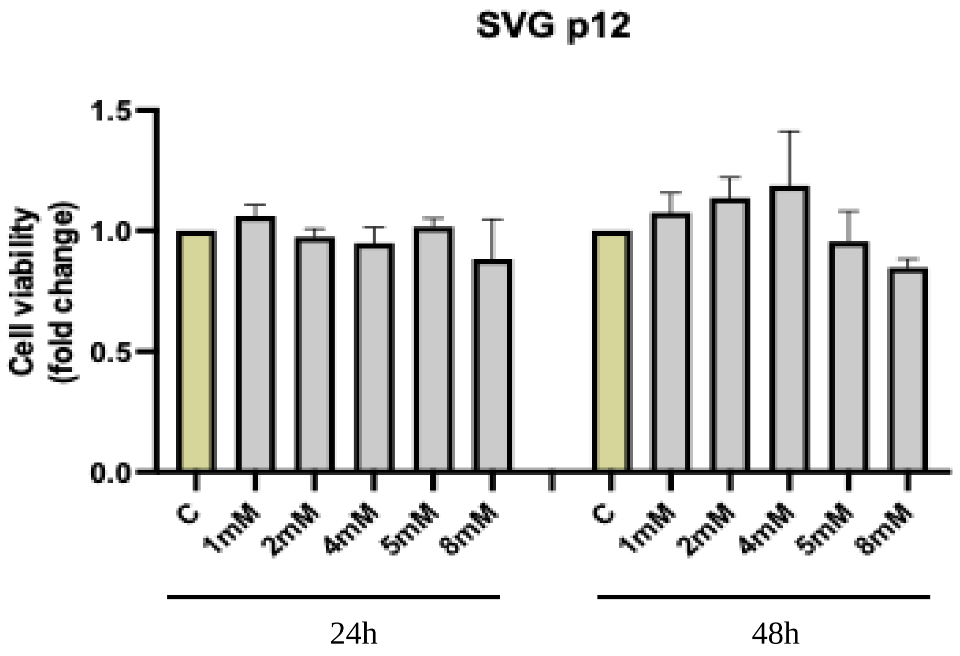

Figure S1. Effects of VA on growth of SVGp12, a human fetal glial cell line derived from normal human astroglial cells. Effect of valproic acid (1, 2, 4, 5 and 8mM), on cell survival in SVGp12 incubated for 24 and 48 h. The data are the mean of n = 3 determinations performed in triplicate. Data presented as mean ± SEM, evaluated by one-way ANOVA test.

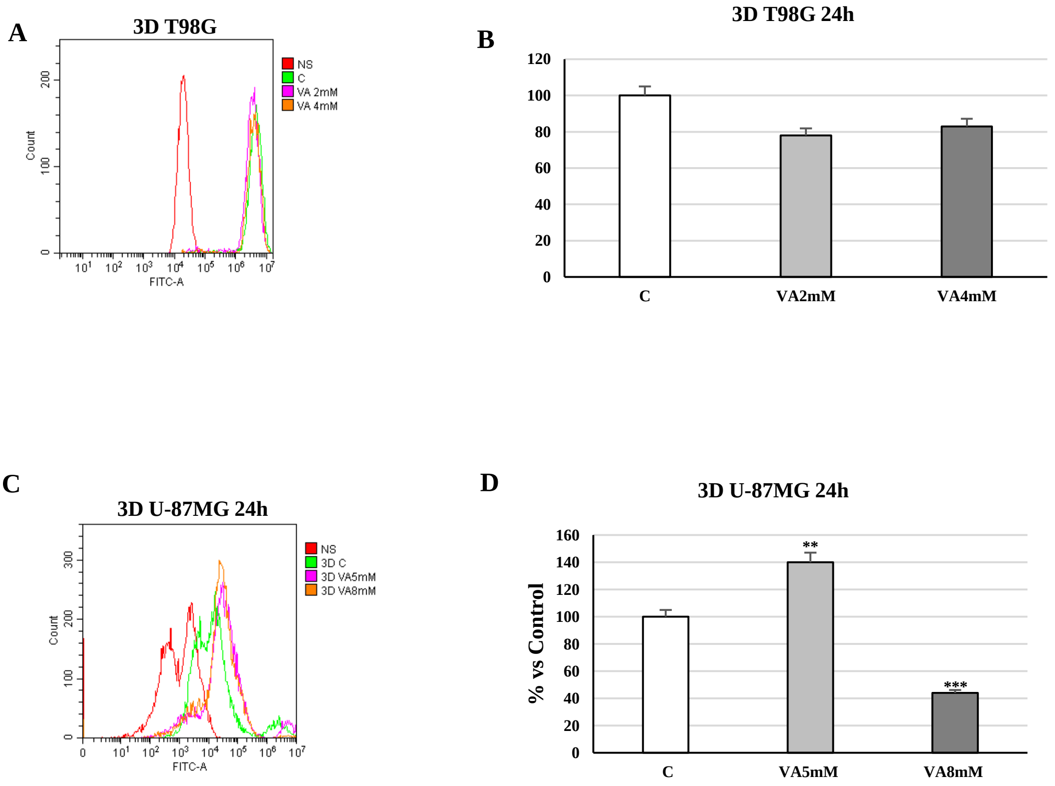

Figure S2. ROS production in VA-treated 3D glioblastoma cells. Intracellular ROS levels were analyzed in 3D T98G (**A**,**B**) and U-87MG (**C**,**D**) cells. Unstained cells (NS) without fluorescent probe were used as negative control. Values represent the mean of three triplicate independent experiments. ** $p < 0.01$, *** $p < 0.001$ vs C.

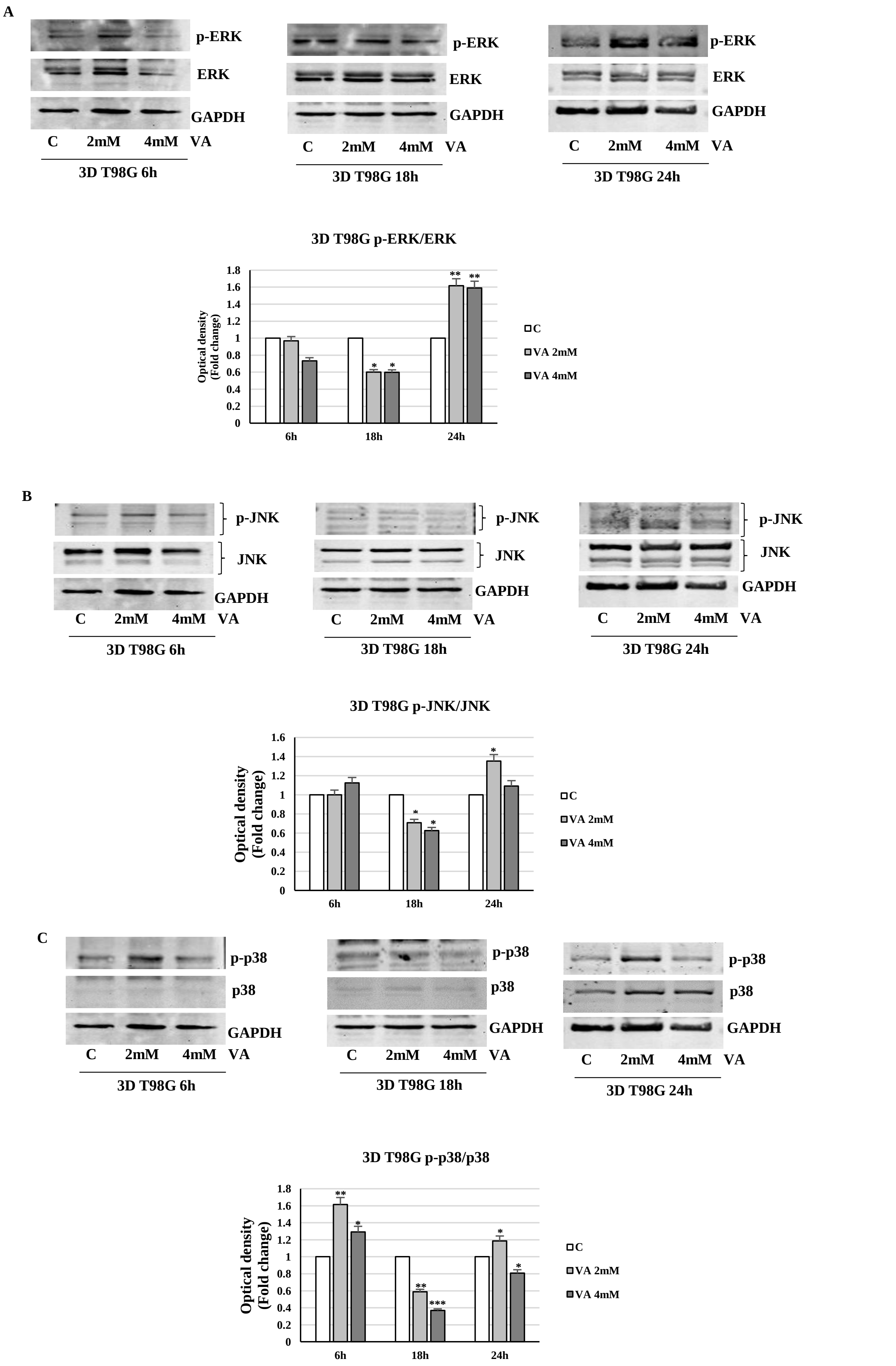

Figure S3. VA influences the MAPK pathways in 3D T98G cells. Immunoblotting of phospho-ERK/JNK/p38 (**A**-**C**) and relative protein levels. The histograms represent the mean ± SD of three experiments in which the band intensities were evaluated in terms of arbitrary units of optical density (OD) and expressed as a fold change relative to the control. * $p < 0.05$, ** $p < 0.01$, *** $p < 0.001$ vs. control.
